# Plasma micronutrient status is improved after a 3-month dietary intervention with 5 daily portions of fruits and vegetables: implications for optimal antioxidant levels

**DOI:** 10.1186/1475-2891-8-10

**Published:** 2009-02-10

**Authors:** Maria Cristina Polidori, Juan-Carlos Carrillo, Pablo E Verde, Helmut Sies, Johannes Siegrist, Wilhelm Stahl

**Affiliations:** 1 Institute of Biochemistry and Molecular Biology I, Heinrich-Heine University, Düsseldorf, Germany; 2 Department of Medical Sociology, Heinrich-Heine University, Düsseldorf, Germany

## Abstract

To explore the effects of increasing fruit and vegetable intake and the resulting effects on levels of circulating micronutrients in a community-dwelling population with an already high consumption of fruits and vegetables, 112 volunteers (86% women) underwent targeted dietary counseling for three months. At the beginning of the study and after 4, 8 and 12 weeks a food frequency questionnaire was filled in, and plasma levels of dietary antioxidants as well as biomarkers of oxidative lipid and protein damage were determined. Compared to baseline, especially the intake of fruits was significantly improved after 3 months of intervention, and mean plasma levels of lutein, zeaxanthin, β-cryptoxanthin, lycopene, α- and β-carotene, retinol, α-tocopherol, vitamin C and vitamin B6 were increased. Biomarkers of oxidative stress remained unchanged. Thus, a nutritional counseling program is capable of improving plasma levels of antioxidants even in a health-conscious population. A decrease in biomarkers of oxidative stress, however, does not occur.

## Findings

Dietary habits are an important instrument of active care for maintaining the population's health due to the association between antioxidant-rich food intake and the occurrence of age-related diseases [1]. A large part of the population, however, does not meet the recommended daily intake of five portions of fruits and vegetables . Research is still needed on micronutrient requirements for optimal health and adequate vitamin intake. Identification of the conditions favoring initiation and maintenance of a healthy nutrition, especially so in adult working populations as target groups of preventive activities, has become a major health priority.

One-hundred twenty-nine employees of a University Hospital in Düsseldorf, Germany, were recruited in the present study. All study participants gave their informed signed consent and followed an intervention plan consisting of 4 two-hour sessions for three months: baseline, T0; 4 weeks, T1; 8 weeks, T2; and 12 weeks, T3. In the sessions, guided by a nutritionist and a physician, participants were motivated to consume at least five portions of fruits and vegetables daily, i.e. at least 400 grams fruits and vegetables per day for at least the whole duration of the study. Participants were asked to fill in every two weeks a standardized qualitative food frequency questionnaire (FFQ) modified from Winkler and Döring [2] as previously described [3]. The nutritional behaviour was evaluated using a nutritional index [2] based upon the dietary guidelines of the German Nutrition Society (Deutsche Gesellschaft für Ernährung, DGE) and participants were divided in three groups: class A = optimal (≥ 5 fruit and/or vegetable portions daily), class B = normal (3–4 fruit and/or vegetable portions daily), and class C = poor (≤ 2 fruit and/or vegetable portions daily) nutritional behaviour.

Blood samples (collected in a heparinized tube and immediately centrifuged), medical history and clinical data including BMI were available at each session. Plasma (or supernatant of deproteinized plasma to preserve vitamin C) was stored frozen at -80°C until analysis. Carotenoids (lutein, zeaxanthin, β-cryptoxanthin, lycopene, α- and β-carotene) were analyzed by HPLC with UV/vis detection [4] with a second UV/vis detector connected in series for quantitation of retinol (vitamin A) and α-tocopherol (vitamin E). Plasma vitamin C levels were measured by HPLC with UV detection, vitamin B6 and malondialdehyde (MDA, a marker of lipid peroxidation) by HPLC with fluorescence detection using commercial kits (Chrom-Systems Instruments and Chemicals GmbH, Munich, Germany). For the measurement of protein oxidation, immunoglobulins G (IgG) were separated from plasma and IgG carbonyls were assessed by ELISA [5]. Statistical analysis was performed with the public domain statistical software R (Development Core Team 2004) version 2.2.0. Two-sided pair-wise t-test was used to measure timepoint-to-timepoint micronutrient changes after correction for age, gender and smoking status. Patients' compliance was assessed with a dynamic migration model on the basis of the probability to move from a poor nutritional class to a better one [2] during the course of the study. Significance was accepted if the null hypothesis was rejected at the p < 0.05 level.

One hundred-twelve subjects (16 M, 96 F, 53.0 ± 9.9 years) with complete data sets from all sessions were included in the final analysis. Twelve% of the subjects were smokers, 73% considered themselves healthy and 27% declared only minor complaints. The distribution of participants into class A, B and C at T0 and at T3 is shown in Table 1. The dynamic migration model showed a good compliance to the intervention including participants with poorer nutrition (p < 0.01). The percentage of class C participants decreased from 25% to 7% in as little as 2 weeks from the beginning of the study (data not shown), and further decreased to 2% after 12 weeks of intervention (Table 1).

**Table 1:** Distribution of participants (%) into class A = optimal (≥ 5 fruit and/or vegetable portions daily), class B = normal (3–4 fruit and/or vegetable portions daily), and class C = poor (≤ 2 fruit and/or vegetable portions daily) nutritional behaviour at time points T0 and T3 (12 weeks).

	Fruit plus vegetable intake			Fruit intake			Vegetable intake		
FFQ	A	B	C	A	B	C	A	B	C
T0	67	8	25	69	25	6	27	46	27
T3	94	4	2	92	5	3	49	34	17

Results extracted from the food frequency questionnaire.

Most of the participants were already following health-conscious lifestyle and baseline micronutrient plasma levels were quite high (yet in the range described in the literature), but a further significant increase of lutein, lycopene, α- and β-carotene, vitamins C and B6 was achieved at T3 compared to baseline (Table 2) (p < 0.01). Even in the absence of a control group not undergoing dietary counseling, it is conceivable that the increase in micronutrients is due to the nutritional intervention. A recent meta-analysis of 38 trials comparing dietary advice with no advice has shown that dietary advice increases fruit and vegetable intake by 1.25 servings/day (95% CI 0.7 to 1.81) as well as plasma levels of selected carotenoids [6].

**Table 2:** Plasma levels of micronutrients and vitamins as well as IgG content of protein carbonyls and plasma MDA levels observed in the study participants (n = 112) at each monthly counseling session (mean ± SD): baseline (T0), 4 weeks (T1), 8 weeks (T2) and 12 weeks (T3).

Parameter	T0	T1	T2	T3
Lutein (μmol/L)	0.37 ± 0.16	0.39 ± 0.16	0.40 ± 0.15	0.41 ± 0.16 *
Zeaxanthin (μmol/L)	0.08 ± 0.07	0.08 ± 0.03	0.08 ± 0.03	0.08 ± 0.03
β-Cryptoxanthin (μmol/L)	0.35 ± 0.36	0.35 ± 0.28	0.31 ± 0.25	0.27 ± 0.19
Lycopene (μmol/L)	0.45 ± 0.28	0.50 ± 0.33	0.49 ± 0.30	0.51 ± 0.28 *
α-Carotene (μmol/L)	0.12 ± 0.13	0.19 ± 0.18	0.19 ± 0.17	0.18 ± 0.17 *
β-Carotene (μmol/L)	0.65 ± 0.45	0.87 ± 0.64	0.86 ± 0.65	0.85 ± 0.62 *
Retinol (μmol/L)	1.35 ± 0.35	1.33 ± 0.35	1.34 ± 0.35	1.33 ± 0.30
α-Tocopherol (μmol/L)	28.9 ± 10.3	28.7 ± 11.4	28.4 ± 9.6	29.5 ± 9.8
Vitamin C (μmol/L)	55.1 ± 20.9	57.4 ± 26.9	55.6 ± 23.3	63.6 ± 21.8 *
Vitamin B6 (nmol/L)	48.3 ± 30.9	65.4 ± 39.2	64.0 ± 37.5	64.4 ± 41.6 *
IgG Carbonyls (nmol/mg)	0.79 ± 0.64	0.87 ± 0.9	0.87 ± 0.79	0.80 ± 0.67
MDA (μmol/L)	0.15 ± 0.11	0.17 ± 0.12	0.16 ± 0.11	0.17 ± 0.16

* p < 0.01, two-sided pair-wise t-test after correction for age, gender and smoking status

Although the levels of micronutrients were increased after 3 months of intervention, no significant change between T0 and T3 was observed both in plasma levels of MDA and the carbonyl content of IgG. This might be partly due to the lack of a significant increase in α-tocopherol levels (Table 2), or to the fact that increasing fruit and vegetable intake for 3 months may not be sufficient to lower plasma MDA and IgG carbonyl content. Our study participants started at levels of these biomarkers compatible with those of a healthy population [3]. Therefore, it is likely that it is not possible to achieve a further decrease of oxidative stress biomarkers in the absence of major illness and in the presence of already low levels. In a 25-day intervention study with either a diet devoid of fruits and vegetables, a diet containing 600 g of fruits and vegetables per day, or this diet plus a vitamin- and mineral-supplement pill, markers of oxidative damage to proteins and lipids did not change after the intervention in either group [7].

The results of this study are encouraging, showing a high compliance to a nutritional counseling program in middle-aged employed women and men resulting in significant increases of plasma levels of several beneficial micronutrients. These results also imply that 1. pronounced health effects of antioxidant-rich diets are to be expected in poorly nourished subjects with likely increased or borderline levels of oxidative stress biomarkers, and that 2. even health-conscious subjects might benefit from long-lasting balanced dietary patterns. Two main conclusions can be therefore drawn from this intervention study. First, the changes in antioxidant micronutrients might at least in part explain the inverse association between high intake of fruits and vegetables and several age-related diseases, and their achievement should be encouraged especially in malnourished populations. Second, attempts should be made to better understand the response of the organism to dietary-induced marked increases in micronutrients, as *in vitro *studies on carotenoids show for instance that, beyond the 'optimum' levels, further increases of carotenoid levels in cells may lead to prooxidant effects [8]. As health-conscious individuals appear to already consume the minimum daily amount of fruits and vegetables suggested by the nutrition societies and accordingly show adequate levels of circulating antioxidant micronutrients, research should focus on establishing definite optimal micronutrient reference ranges.

## Competing interests

The authors declare that they have no competing interests.

## Authors' contributions

MCP, JS, HS and WS conceived and designed the study. MCP carried out the nutritional sessions and clinical assessments including blood and FFQs collection. MCP and JCC performed all analytical measurements. PEV and JS created the database and provided data analysis. MCP, JS, HS and WS interpreted the results and drafted the manuscript. JS and WS have given final approval of the version to be published. All authors read and approved the final manuscript. JS and WS supervised the work equally.

## Authors' information

HS is a Fellow of the National Foundation for Cancer Research (NFCR), Bethesda, MD. MCP is currently Fellow of the Forschungskolleg Geriatrie, Robert Bosch Foundation, Germany.
